# Engagement of patients and the public in personalised prevention in Europe using genomic information: a scoping review

**DOI:** 10.3389/fpubh.2024.1456853

**Published:** 2024-09-12

**Authors:** Loes Lindiwe Kreeftenberg, Lidewij Henneman, Johannes C. F. Ket, Martina C. Cornel, Carla G. van El

**Affiliations:** ^1^Department of Human Genetics, Amsterdam UMC, Vrije Universiteit Amsterdam, Amsterdam, Netherlands; ^2^Amsterdam Public Health Research Institute, Amsterdam, Netherlands; ^3^Medical Library, Vrije Universiteit Amsterdam, Amsterdam, Netherlands

**Keywords:** patient engagement, public engagement, empowerment, personalised prevention, personalised medicine, genomics, precision prevention, chronic diseases

## Abstract

**Introduction:**

Personalised prevention using genomic information requires active involvement from patients and the public, who should be well-informed and empowered to make healthcare decisions that reflect their personal values. We aimed to map engagement practises, and assess the extent and types of engagement methods used in the field of personalised prevention of common chronic conditions using genomic information.

**Methods:**

A scoping review on selected literature (in Medline, Embase, Scopus, Web of Science, APA PsycINFO, and IBSS) from 2015 to 2023 was performed. Articles included described practises of patient and public engagement in personalised prevention and genomics conducted in Europe focusing on cancer, cardiovascular diseases and neurodegenerative disorders. Engagement was explored based on grouping practises across the domains of care, research, education, and governance.

**Results:**

A total of 23 articles describing 23 engagement practises were selected. Analysis revealed diverse engagement levels, the majority falling into the low to medium engagement category, and showing mainly unidirectional methods of engagement, especially consultation. Most engagement activities related to cancer, and none to neurodegenerative disorders. Most publications appeared in the care domain, followed by the research domain, a combination of research and care, and a combination of governance and education.

**Conclusion:**

These results suggest that most practises to engage patients and public in personalised prevention using genomic information appear to have lower levels of engagement. Elaborating on and implementing practises that engage and empower patients and the public at all levels of the engagement spectrum and for all chronic diseases is needed, fostering a more inclusive and participatory approach to personalised prevention.

## 1 Introduction

With the burden of chronic diseases growing and the population ageing, prevention has become paramount. The transition in public health and healthcare from “one size fits all” to “person-centred” disease prevention and early diagnosis has been suggested to foster the implementation of sustainable and high value healthcare (1, 2). Chronic conditions are a key focus of personalised prevention, as they currently affect one-third of adult European Union (EU) citizens (3), and lead to 900,000 premature deaths annually in EU countries (4).

In recent years a new vision of personalised prevention has emerged as a specific focus within personalised medicine, in which genomic information plays an important role and ensures more precise prevention, diagnosis, and treatment of diseases by integrating information on individuals’ omics (genetics, proteomics) (5). In light of the growing body of genome-based knowledge and modern advances in biomedicine, there is a need for a conceptual shift in public health. These advancements allow healthcare systems to adjust their preventative strategies due to a better understanding of disease causation and pathways (6). Personalised prevention has been defined as aiming to “prevent the onset, progression and recurrence of disease through the adoption of targeted interventions that consider the biological information (e.g., genetics, demographics, health condition), environmental and behavioural characteristics, the socio-economic and cultural context of individuals” (7). Personalised prevention is thereby closely linked to research as knowledge is constantly being developed by processing large quantities of data on biology, environment, and behaviour (8).

A personalised approach to healthcare requires patients and the public to be engaged in healthcare and public health, which implies being well-informed, knowledgeable about data sharing, and empowered to make decisions that reflect their personal values (9, 10). In this study, empowerment, at its core, refers to individuals’ ability to take action and control of their own health and their right to actively participate in any decision-making that affects them (11), ensuring that their personal values and preferences are reflected in their healthcare and public health choices. A well-informed patient is prepared for shared decision-making, equipped with a thorough understanding of their condition, intervention choices, and lifestyle implications, fostering a collaborative and patient-centred approach to healthcare and public health (12).

Public and patient engagement is multi-faceted, terminology varies, especially across international settings and depending on the specific application (13). Levels of engagement may vary from informing (e.g., website, webinar, workshop) to placing decisions in the hands of the public (14). If the public, patients and their families are to be active partners, it can be argued that they must be systematically and meaningfully engaged in the planning, delivery and evaluation of personalised prevention practises. Engagement can be explored across various domains relevant for personalised prevention: being involved in decision making regarding one’s health, benefiting from or contributing to research, having adequate knowledge, and responsible governance (13). Therefore, this study focuses on the domains of care, research, education, and governance as a key organisational framework to explore engagement.

In the care domain, public and patient engagement aims to increase the active participation in healthcare (10, 15). Individuals should feel empowered to make health decisions that align with their personal values and preferences which leads to more culturally sensitive and patient-centred care. When patients feel heard, and are actively involved, they are more likely to take ownership of their health, engage in preventive measures, and adhere to recommended interventions (9).

In the research domain, patients with personal experience of a disease offer a unique perspective that, if explicitly incorporated, leads to science that is more relevant and translatable (16). The term patient engagement in research has been used to characterise patient and public contributions to research via roles that range from “passive” study participants to “active” patients and public involved in all phases of research (17).

With regard to the education domain, it is critical for the general public and patients to have a clear understanding of their health risks and the potential to lower those risks through lifestyle modifications or other preventive interventions (18, 19). Individuals should be well-informed about the potential benefits that can be derived from the integration of genomics into healthcare, as well as being aware of potential challenges or limitations that may arise. Public and patients engagement in this domain also includes involving these stakeholders in the development of educational materials, training programmes, and patient education resources (20).

Lastly, the governance domain emphasises the involvement of the public and patients in decision-making processes regarding personalised prevention policies and programmes, encompassing their participation in policy development, guideline formulation, and organisational governance structures. Literature has shown that trust in scientific and medical institutions is concomitant with stakeholder engagement. In order to implement personalised prevention and genomics in the EU and gain the patients’ and public’s trust to contribute health data to science, stakeholders must be involved throughout the policy cycle (21).

The engagement practises can concern any of the levels primary, secondary or tertiary prevention, where primary would refer to preventing the onset of symptoms, secondary the early identification and intervention, and tertiary the treatment avoiding further consequences (22).

This scoping review aims to explore aspects of patient and public engagement in the field of personalised prevention and genomics across common chronic conditions. This review intends to comprehend what kind of engagement practises exist in Europe to understand in what ways and to what extent citizens and patients are currently engaged, how such practises may relate to the concept of empowerment and to identify gaps for improvement (23).

Public and patient engagement in the field of personalised prevention is an integral part of the EU project “A PeRsOnalised Prevention roadmap for the future HEalThcare (PROPHET)” (7). This scoping review is contributory to the PROPHET project, which aims to co-create a Personalised Prevention Roadmap for the future healthcare with stakeholders, in order to support the definition and implementation of innovative, sustainable and high-quality personalised approaches that are effective in preventing chronic diseases (7).

## 2 Methods

A scoping review was conducted to systematically map the European public and patient engagement practises in the field of personalised prevention and genomics for common chronic diseases. We reported this scoping review in accordance with the Preferred Reporting Items for Systematic reviews and Meta-Analyses extension for Scoping Reviews (PRISMA-ScR)-checklist (see Supplementary Data Sheet 1) (24). Patients and the public were not involved in this study, but input was sought from the Active Citizenship Network (ACN) and the European Patients Forum (EPF) on main concepts.

### 2.1 Eligibility criteria

According to the PICO framework, the research question and the requirements for inclusion in our study were developed.

Population: patients and the general public in Europe being engaged in personalised prevention and genomics across common chronic conditions.

Intervention: engagement practises: e.g., survey, webinar, interviews, workshop, focus groups, apps, games, capacity-building, forums, dialogue ongoing or concluded in 2023.

Comparator: not applicable.

Outcome: Consultation, collaboration or patient/public-directed engagement practises in the field of personalised prevention.

Publicly accessible publications in the English language with regard to public and patient engagement practises in the field of personalised prevention and genomics were deemed eligible according to the PICO framework. Practises based in Europe were the focus as this is a contributing article to a European project: PROPHET. The timeframe was limited to articles published between 2015 and August 2023 in order to display an up-to-date image of the state of the art of the existing engagement practises. We restricted our search to documents that were published in 2015 or later, since the EU Health Ministry’s first defined personalised medicine in the Council Conclusions on Personalised Medicine for Patients in 2015 (25). In addition, in its 2015 report titled “Shaping Europe’s Vision for Personalised Medicine,” the EU-funded project “PerMed” listed raising awareness and empowering patients as one of the five challenges of personalised medicine (26). Following the focus of the PROPHET project, the selection of studies are specifically related to engagement practises in the field of cancer, cardiovascular diseases and neurodegenerative disorders. Rare metabolic/genetic hereditary diseases with a low prevalence (<1 per 2000) are excluded (27), as mandated by the project’s emphasis on “predominantly prevalent chronic diseases.” However, we include monogenic sub forms of such diseases. Animal research and environmental studies were excluded. Furthermore, if a publication did not include a combination of the following elements: genetics/genomics, prevention, engagement, and a chronic condition, it was eliminated.

### 2.2 Information sources and search strategy

A comprehensive search was performed in the databases: OVID/Medline,^1^ Elsevier/Scopus, Clarivate Analytics/Web of Science Core Collection, Ebsco/APA PsycINFO and Proquest/International Bibliography of Social Sciences (IBSS), from 2015 to July–August 2023 (see Supplementary Data Sheet 2 for exact dates) in collaboration with a medical information specialist (JCFK). The search included controlled terms and free text terms for synonym terms for “public,” “patient,” “stakeholder,” “community” in combination with “personalised prevention,” “personalised medicine,” “genetics,” “genomics” and “engagement,” “participation,” “information,” “consultation,” “involvement,” “collaboration,” “empowerment” in the following three disease groups: “cancer,” “cardiovascular diseases” and “neurodegenerative diseases.” The search was performed without restrictions for methodology or language. The search was limited to publication date starting from 2015. Duplicate articles were excluded by a medical information specialist (JCFK) using Endnote X21.0.1 (Clarivatetm), following the Amsterdam Efficient Deduplication (AED)-method (28) and the Bramer-method (29).

This search strategy resulted in 7,317 records. See the Supplementary Data Sheet 2 for the full search strategies per database.

### 2.3 Syntheses of results

Publications were clustered according to the disease category and their type of public and patient engagement methods, with the use of a worksheet-based Excel model. The data synthesis process was conducted by a single researcher (LLK) using the Rayyan software, while another researcher (CvE) assessed an independently selected sample of 25% of the articles in the Rayyan software. In cases of disagreement, the two researchers consulted each other to resolve any discrepancies, and if consensus could not be reached, two additional researchers (LH and MC) were consulted for additional input. The pertinent passages from the selected publications were allocated to the Data Extraction Sheet, utilising an Excel worksheet-based model (Supplementary Table 1). For each publication and corresponding engagement practise, the engagement modalities, including the engagement method, engagement domains, and the extent of engagement and stage of prevention were assigned.

### 2.4 Data items

#### 2.4.1 Extent of engagement

**Figure 1:**
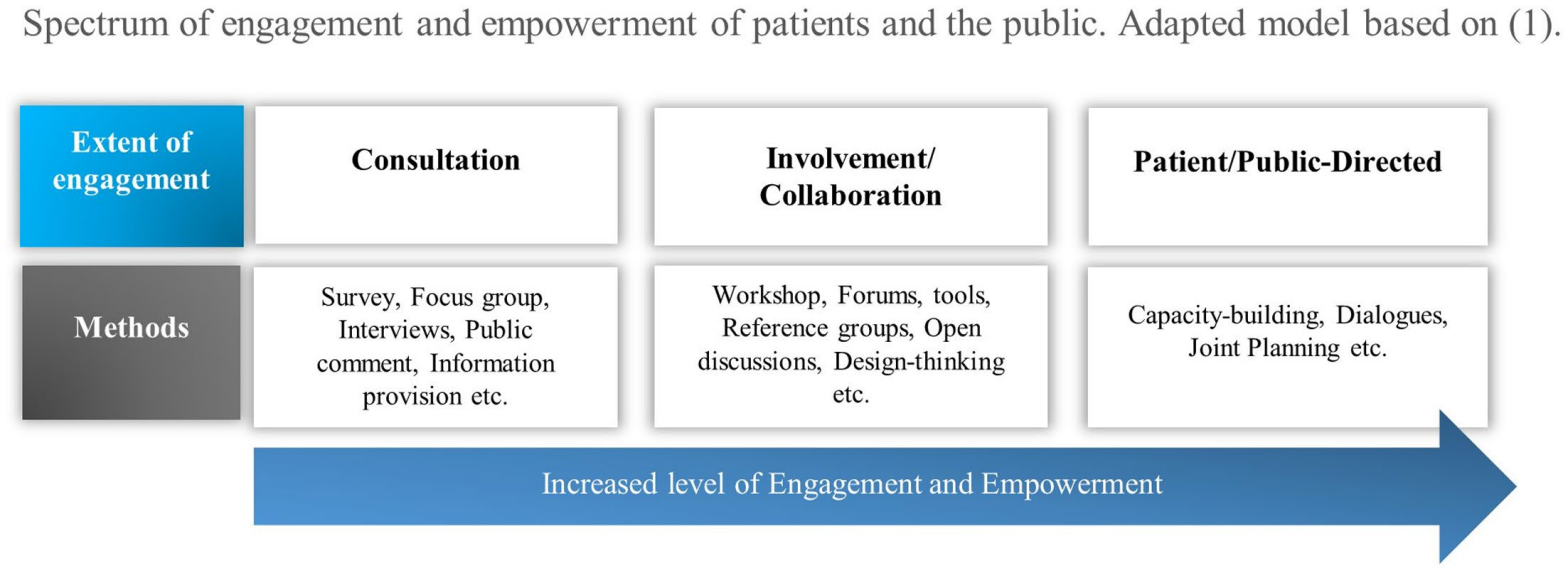
Spectrum of engagement and empowerment of patients and the public. Adapted model based on IAP2 and Shimmin (14, 73).

To analyse the level of public and patient engagement in the identified engagement practises, we used a modification of the International Association of Public Participation (IAP2) spectrum of public participation adapted by Shimmin (14, 30) (Figure 1). We extended the use of the revised Shimmin model beyond research to include the domains care, education and governance. This study utilises these domains as a key organisational framework to explore their interplay in the context of personalised prevention and healthcare systems.

The adapted model introduces three distinct levels or modes of engagement and empowerment: Consultation, involvement/collaboration, and patient/public-directed. At the Consultation level (low level of engagement), the primary objective is either to provide information or to gather feedback, input from patients and the public. We merged the categories of the Shimmin model “Involvement” and “Collaboration” because we found significant overlap in the descriptions and outcomes of the engagement practises. Involvement/Collaboration (medium level of engagement) involves an ongoing partnership where decision-making is shared between stakeholders (higher level of engagement). Lastly, Patient/Public-Directed is referred to as “user-controlled” or “user-led,” where patients and/or members of the public play a central role in decision-making (high level of engagement) (14).

## 3 Results

**Figure 2:**
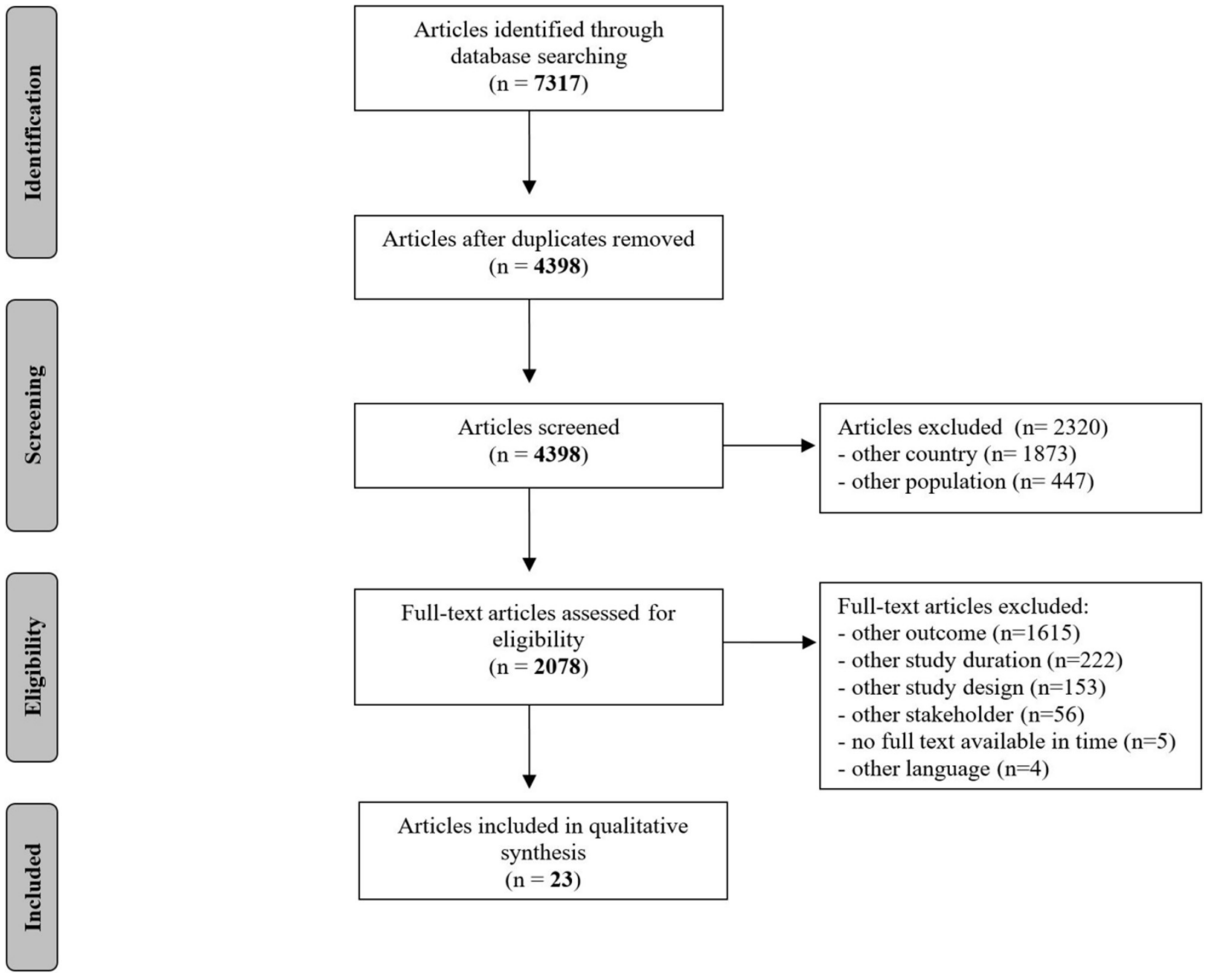
PRISMA 2020 flow diagram for the selection and screening process in the scoping review.

Figure 2 displays the screening and selection process of the scoping review. The total number of 7,317 records was reduced to 4,398 by removing duplicates. The abstract screening process resulted in 2,320 titles being excluded as irrelevant, with 2,078 articles remaining for full-text-screening. During full-text-screening we removed 1,615 publications due to the content or outcome of the publication, or the source of information as described in our exclusion criteria, such as when there is no genomics/genetics involved or no focus on prevention of chronic conditions. The publications with other study designs (*n* = 153), including case-reports and systematic reviews were removed to ensure the inclusion of only primary studies. Additionally, articles with other time periods (*n* = 222) were removed. In 56 publications a very heterogeneous sample was examined: healthcare professionals, policy makers but also representatives of other groups that did not meet our inclusion criteria, and thus were excluded. The entire article selection procedure resulted in 23 engagement practises described in 23 articles for this review that were published between 2015 and 2023 (see Supplementary Table 1).

### 3.1 Description of engagement practises

The majority of the engagement practises were targeted to patients (10; 44%) (31–40). Three practises focused on patient representatives (13%) (37, 41, 42), one on the combination of patients and families (4%) (43), one practise targeted both patients and the public (44), and eight involved public engagement practises (35%) (45–52). Most practises (18; 78%) focused on (hereditary) cancer, specifically breast cancer and ovarian cancer (31–39, 42–44, 46–48, 51–53). Cardiovascular disease engagement practises were reported in two practises (9%) (40, 41). No engagement practises focused on neurodegenerative disorders. Three practises (13%) focussed on personalised prevention or personalised medicine in general (45, 49, 50).

Four of the identified engagement practises (17%) were conducted in the United Kingdom (31, 38, 46, 47), four (17%) in France (32, 37, 42, 51); three (13%) in Germany (36, 39, 52); two (9%) in Switzerland (45, 50); two (9%) in Italy (44, 53); two (9%) in Denmark (43, 49); two (9%) in the Netherlands (35, 41); two (9%) in Estonia (34, 40). One engagement practise (4%) was conducted in Sweden (48); and one (4%) in Belgium (33).

#### 3.1.1 Extent and methods of engagement

**Figure 3:**
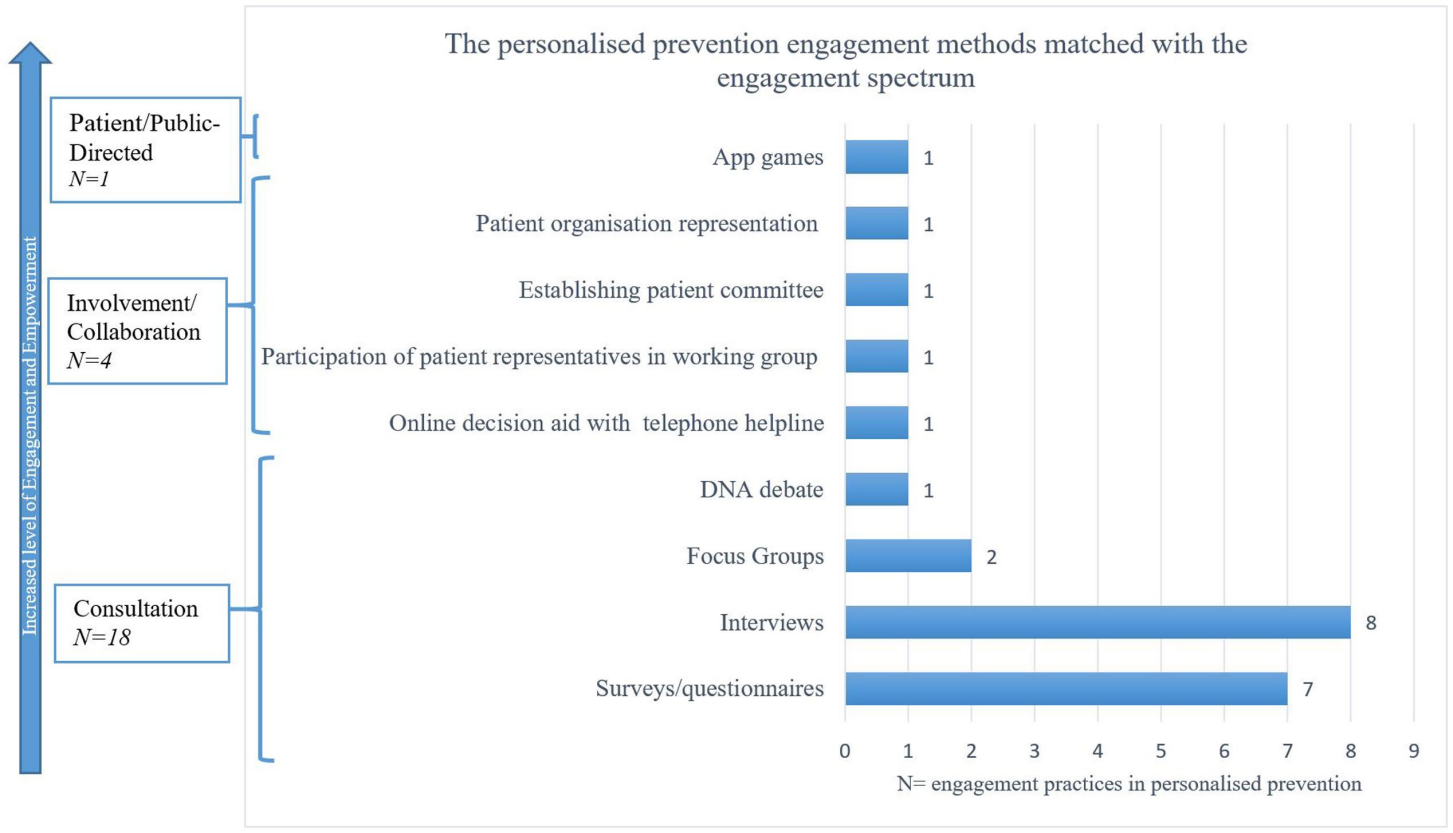
The personalised prevention engagement methods matched with the engagement spectrum.

Figure 3 displays the different levels and extents of engagement. Displaying that the majority (18/23; 78%) of the practises were found at the Consultation level of the engagement spectrum, by generally asking for input or opinions at set points in the process of personalised prevention and genomics (unidirectional) and not providing an ongoing opportunity for input. The methods used included surveys (32, 34, 40, 44, 49–51); interviews (31, 35, 36, 38, 39, 41, 43, 47); focus groups (45, 48) and a mix of a focus group with a DNA debate (33) (Figure 3). Four engagement practises were reported at the Involvement/Collaboration level (17%). Methods included patient organisation representation (53); online decision aid tools with an optional helpline (46) as well as participation of patient representatives in working groups (37) and establishing patient committees (42). One practise (4%) was reported at the Patient/Public-directed level which included empowerment app games which were co-created with citizens (52), by building on their feedback as well as engaging the public in the design and assessment of the digital game. The minority of practises (5/23) were found on the higher level of the engagement spectrum. Supplementary Table 1 shows more extensive information of the “engagement modalities” of each practise.

### 3.2 Primary, secondary, and tertiary prevention

**Figure 4:**
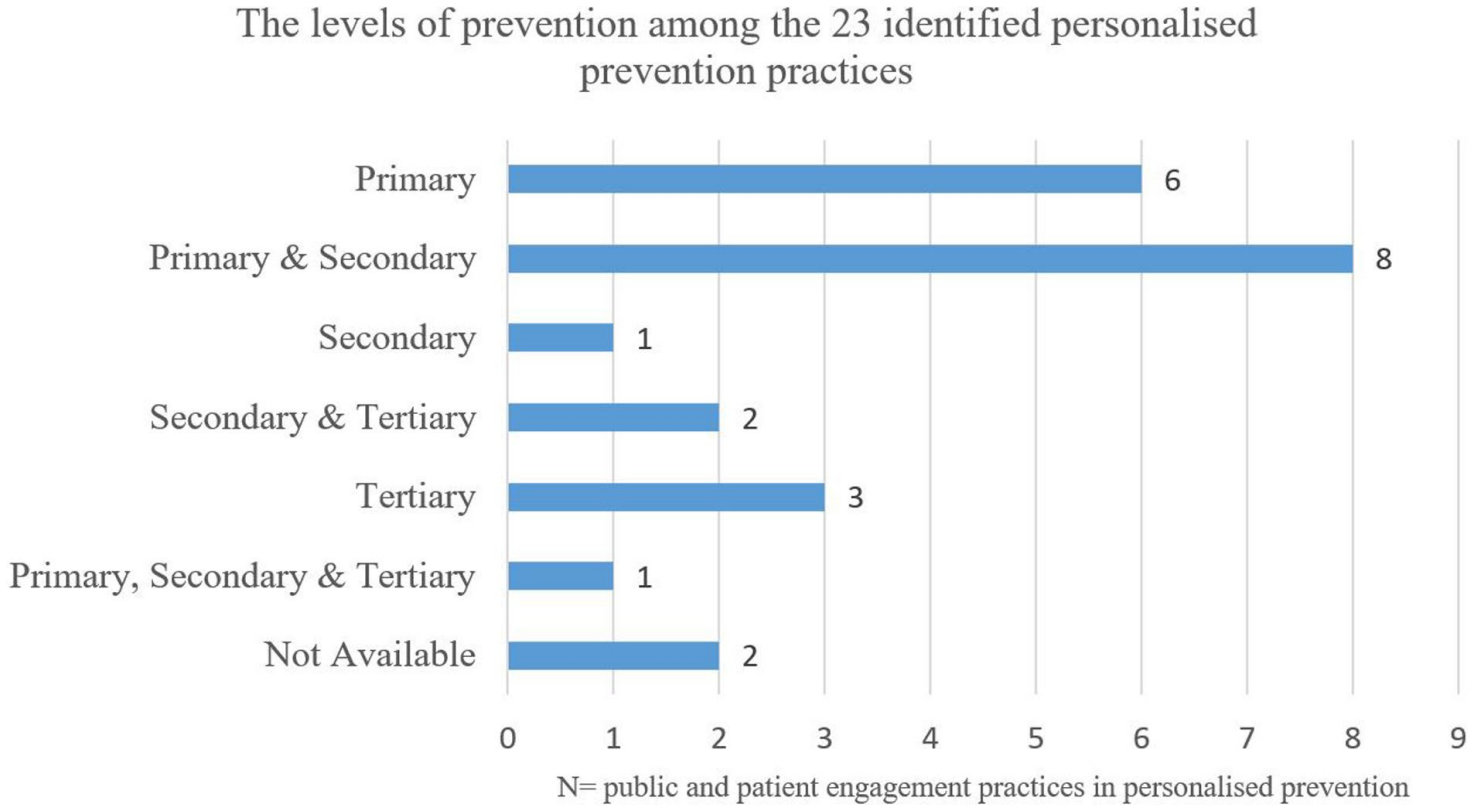
The levels of prevention among the 23 identified personalised prevention practice.

Figure 4 presents an analysis of the categorisation of personalised prevention engagement practises across the different levels of prevention. The majority of practises fall into one or two categories; six practises (26%) are classified as primary prevention (32, 40, 45–47, 51), eight practises (35%) categorised as primary and secondary (33, 34, 37, 39, 48–50). One practises (4%) is categorised as secondary (33, 44). Furthermore, two engagement practises (9%) categorised as secondary and tertiary (31, 53), three practises (13%) are categorised as tertiary (35, 36). It reveals that one practise (4%) falls into all three categories: primary, secondary, and tertiary prevention. In this example, the parents’ perspective on paediatric cancer families’ participation in whole genome sequencing (WGS) research were studied in Denmark. It is classified as primary prevention, as WGS may identify potential genetic predispositions for diseases unrelated to the original indication (the first cancer diagnosed) which could lead to interventions that prevent the onset of a disorder (especially other cancers). We also classified this practise as secondary and tertiary prevention because it focuses on paediatric cancer early diagnosis and interventions that may prevent disease progress (43). Two practises (9%) in the research domain were not clearly classified into a single category, or the information provided was insufficient to determine the appropriate classification (42, 52).

### 3.3 Domains of engagement

**Figure 5:**
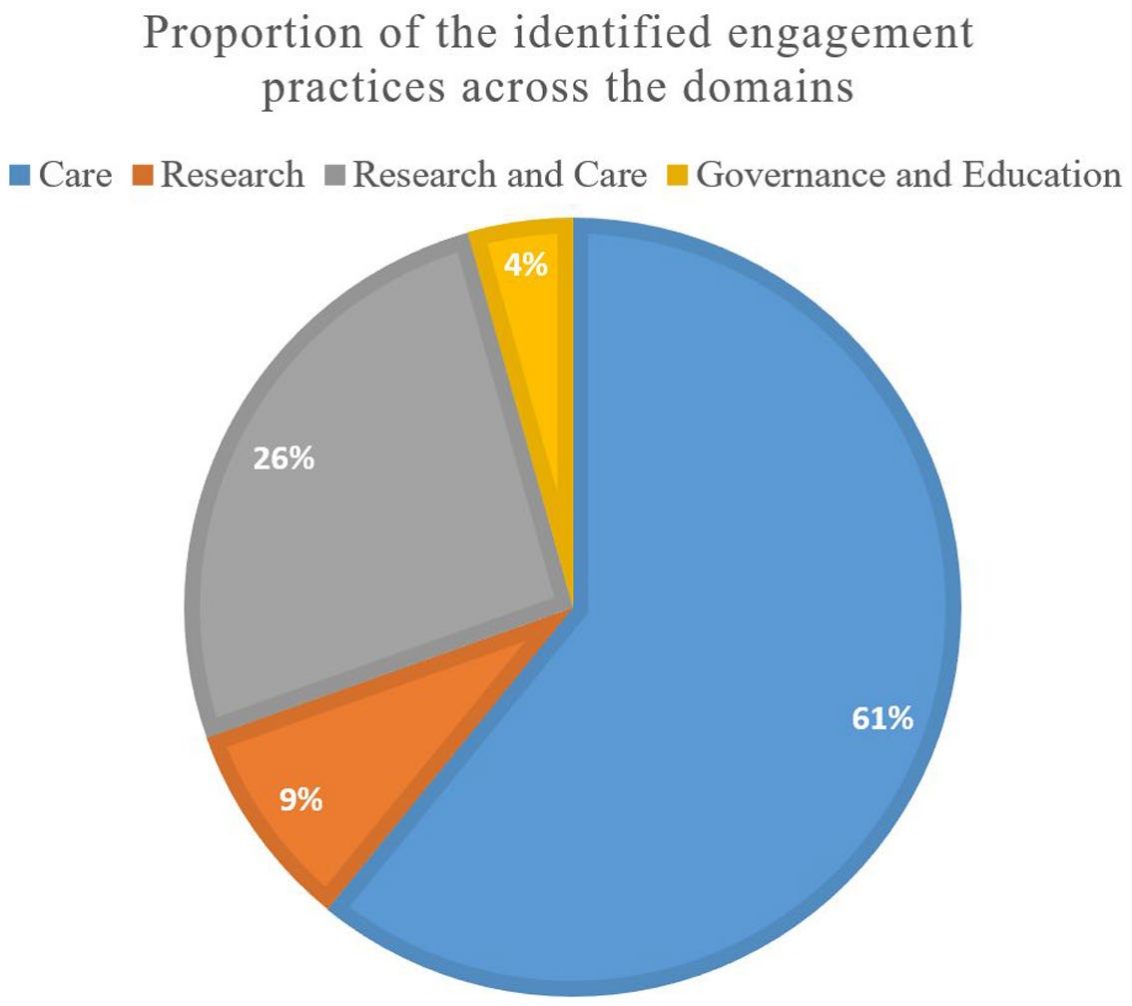
Proportion of the identified personalised prevention engagement practices across different domains.

Figure 5 distinguishes the 23 engagement practises across the four domains: care, research, education and governance. The majority of the practises were found in the care domain (14; 61%), two in the research domain (9%), six practises in the research to care domain (26%), and one in the domain of governance combined with education (4%).

#### 3.3.1 Care domain

Eight out of the 14 engagement practises in care were targeted at patients or patient representatives; five practises focused on the public. One exploratory analysis focused on breast cancer patients as well as healthy subjects (44).

Five practises used interviews as a means to achieve patient or public engagement (35, 38, 39, 41, 47). Fifteen patients with colorectal cancer participated in semi-structured interviews, designed to get their opinions on the dissemination of information about dihydropyrimidine dehydrogenase (DPD) testing (31), that is done before delivery of treatment to reduce side effects. DPD deficiencies associated with variants in the dihydropyrimidine dehydrogenase gene (DPYD) are frequently related to severe side effects after fluoropyrimidine chemotherapy. The engagement activity aimed to evaluate testing acceptance in standard clinical care (35).

Three practises focused on asking for feedback with the use of surveys (32, 40, 44, 49). An example of a survey with a group approach to genetic counselling for hereditary breast and ovarian cancer amidst rising referrals, was evaluated to assess if this would not compromise care. Patients’ awareness of cancer genetics, genetic testing, and the significance of the results improved after attending the group session (32).

One practise used focus groups as a tool to investigate members of the Swedish public’s attitudes and preferences for receiving genetic risk information about hereditary cancer risk (48). Through focus groups and a debate, cancer patients’ opinions, concerns, and expectations about using Next Generation Sequencing (NGS) in genetic diagnostics were collected (33). A total of 1,250 contributions were obtained from the focus groups with patients and DNA debate sessions with citizens. The topics included privacy concerns, personalised medicine uncertainties, and data governance. Another engagement practise by Pujol et al. (37), focused on enhancing the harmonisation and quality of information conveyed to patients when reporting secondary findings of genome sequencing in cancer. Their engagement practise aimed to formulate guidelines on patient information and consent, and to create informed consent forms along with an informational media tool in the form of an animated movie. This is an example of a high level of engagement and empowerment of patients, as the participation of patient representatives in working groups provided the materials, educational guidelines, and ethical reflection (37).

A feasibility study aimed to evaluate the acceptance, satisfaction, psychological wellbeing, and quality of life of women undergoing population genetic testing risk stratification using an online/web-based decision aid and an optional telephone helpline (46). One practise focussed on an elaborate way to inform women about their risk for breast cancer impacting treatment and prevention options (51). The goal was to give patients personalised risk assessments and screening recommendations tailored to the specific risk levels of each individual, ensuring a more personalised and targeted approach to breast cancer prevention. The authors concluded that while a pathway session with a radiologist, nurse navigator and breast specialist might not be practical in every hospital setting, such risk assessment clinics would make the shift from treatment-oriented care towards early diagnosis, prevention and health promotion.

#### 3.3.2 Research domain

One practise engaged the public and another patients in the research domain. Both practises exemplify high levels of engagement and empowerment, with one engagement practise classified as Collaboration and the other as Patient/public-directed.

The study “Partnering with Patients in Translational Oncology Research: Ethical Approach” (42), underlined the necessity of creating a long-term collaboration and promoting a common understanding among all parties involved. To do this, a patient committee was formed to increase the involvement of patient advocates, in addition to health professionals, in the development of the translational research programme. Patient representatives became full participants in this method, including, actively participating in knowledge dissemination to the public through conferences and publications. This study sought to improve the integration of patient expectations and to ensure a collaborative and inclusive approach in translational oncology research (42).

The engagement practise of GENIGMA, an app designed for mapping the genome of cancer cell lines via citizen science, stands out as the highest form of engagement and empowerment in this study. This patient and public-directed example is a digital game co-created with the public and allows them to actively participate in producing data that surpasses the capabilities of artificial intelligence. The procedure entails presenting the concept to the public, acting on their suggestions, and incorporating them in creating and evaluating the game (52).

#### 3.3.3 Research and care combination domain

Six engagement practises could be regarded as a combination of research and care. Two practises were targeted at the public; three engagement practises were focused on patients and one was focused on the combination of patients and the family.

All six identified practises engaged public and patients at the Consultation level of the engagement spectrum, by generally asking for input at one moment, and not providing an ongoing opportunity for input. Engagement methods found in the research and care domains were interviews (*n* = 3) and surveys (*n* = 2) and a combination of focus groups with interviews (*n* = 1). The latter engagement practise explored perspectives of the general public on a hospital-based biobank aimed at supporting biomedical research, including genomics and personalised medicine. The results emphasised the ethical, social, and policy concerns related to disclosing data in biobanks that employ a broad consent model utilised for in-hospital biobank recruitment. Additionally, participants expressed a desire for more training in genomics and further information regarding the biobank effort (45).

An example of a study that used interviews to gather information about patient motivations for taking part in personalised cancer research focused on the patients’ misconceptions about participating in a clinical trial that was aimed to study stratification (36). Semi-structured interviews were conducted with colorectal cancer patients involved in a biomarker trial for (neo)adjuvant treatment to analyse their viewpoints and comprehension of research and treatment (care). Based on the research findings, patients were found to be only partially aware of the main goal of personalised cancer research, which was to stratify responders and non-responders. The authors advocated for clinicians to be sensitive to possible misunderstanding in informed consent procedures and call for more training and the development of alternative measures to help improve such procedures (36).

#### 3.3.4 Governance and education domain

In the domain of governance and education, the patient engagement article was by the Alliance Against Cancer (ACC), a network of Italian cancer centres dedicated to bridging research and care, which displays a high level of engagement and empowerment in the Collaboration level. Through patient organisation representation, such as by the Italian Cancer Patients’ Organisation (AIMaC), the AAC states that they ensure a bidirectional exchange of information between patients and institutes. This collaboration aims to develop cost-effective processes, provide tailored information on concepts of contemporary personalised and precision medicine to cancer patients to meet the increasing demand for information (53).

## 4 Discussion

This scoping review mapped public and patient engagement practises in the area of personalised prevention and genomics across the following common chronic conditions: cardiovascular diseases, cancers and neurodegenerative disorders. Most engagement activities were related to (personalised prevention of) cancer, and none to neurodegenerative diseases. The results show the variety of practises and approaches that involve patients and the public at different stages of the engagement and empowerment spectrum in personalised prevention. Mainly unidirectional engagement methods were used (consultations such as surveys). It is apparent that on the low end of the engagement spectrum there are few opportunities for individuals to provide feedback on the practise. It is important to note that the bulk of engagement and empowerment practises in this study consulted patients, and only a handful of practises consulted the public. Regarding the prevention level, it is notable that the majority of practises are categorised as primary and secondary prevention. The findings also showcase that the majority of engagement practises were found in the care domain and the minority in the governance domain. Lastly, the majority of practises on the higher level of the engagement spectrum were published between 2020 and 2023.

There is a promising prospect for the future as the emergence of engagement and empowerment practises has increased over time. International initiatives, such as the foundation of the International Consortium for Personalised Medicine in 2016, may have stimulated countries to develop national plans related to genomics and personalised medicine (54). Therefore, it is likely that national and European authorities are becoming more interested in personalised medicine and personalised approaches to health. This has coincided with an increase in the number of (published) patient and public engagement practises in the EU in recent years (10).

The disease focus across the various engagement practises was primarily on cancer, specifically (hereditary) breast cancer, including engagement practises based on breast cancer risk assessment initiatives with the general public, while neurodegenerative disorders, according to our results, have received comparatively little attention. This is remarkable given their acknowledged significant relevance for healthcare systems, as highlighted, for instance, by Nielsen and Boenink (55), who critically looked at conditions for patient involvement in Alzheimer’s biomarker research and beyond. Regarding responsible research and innovation, patient participation is promoted as essential for societal and ethical reflection, before new biomedical technologies are developed. It is mentioned that biomarker research raises several social and ethical issues, and it is not always evident that patients would appreciate these technologies (55). One explanation for the majority of practises focussed on breast cancer is due to the available prevention options and the fact that testing for hereditary cancer (e.g., *BRCA1/2* genes) is available for a number of decades.

The results show various forms of engagement methods across the spectrum of engagement throughout the four domains of care, research, education and governance. A variable degree of public and patient engagement practises was undertaken in the care domain, ranging from simpler open discussions and one-way communication such as surveys and interviews to participation of patient representatives in working groups as a more collaborative two-way communication. The majority of practises were found in the care domain, followed by the research domain. In the research domain, although only a few engagement practises were found—categorised as collaboration and as patient/public-directed— these showcase high levels of empowerment. Many engagement practises were categorised in both care and research due to the blurring of lines between these domains. With respect to governance of personalised prevention, only one practise was found in this study (53). This practise ensured a bidirectional exchange of information between patients and institutes as well as the focus on tailoring the information to the patients. More engagement with the public and patients during the various stages of the public health policy cycle is a crucial element for implementation and may help foster public trust of genomics initiatives in the EU (21, 56). By involving patients and the public in decision-making processes, research and prevention strategies are tailored to the participants’ needs, preferences, and circumstances, ultimately leading to improved health outcomes and a more sustainable healthcare system.

### 4.1 Higher levels of engagement: the higher the better?

More intensive collaboration on patient engagement and empowerment can be established as we saw in the case of Pujol et al. (37), Mamzer et al. (42), and De Paoli et al. (53), through establishing more enduring forms of participation by inviting patients or patient representatives in, e.g., working groups or committees. By involving patients and the public more structurally in decision-making processes, across various domains, their unique perspectives and needs can inform policies and practises. This fosters a sense of ownership and responsibility, empowering patients mostly represented by patient organisations to actively participate in healthcare decisions.

However, there are potential challenges and limitations associated with more high-end engagement practises including patient/public-led or patient/public-directed engagement and empowerment in personalised prevention. One challenge is ensuring representativeness or inclusion of diverse patient or public experiences, as not all individuals may have the resources or ability to participate actively (57). Patient organisations normally represent a broader perspective which encompasses a variety of individual experiences; however awareness is important that engagement approaches, if not carefully implemented, may exacerbate existing inequalities. Some groups and individuals may be more in need of engagement than others, and “one size” is not likely to fit all needs (58). Additionally, balancing patient and public perspectives with scientific evidence and professional expertise can be complex, requiring well-informed “expert patients,” considering the innovation’s nature, the professional and patient characteristics involved, and the social, organisational, political, and economic landscape (59). Programmes that provide education and training to enable patients to meaningfully participate are essential in this sense (60). Furthermore, the scalability and sustainability of more intensive engagement activities may be limited, requiring ongoing support and resources from the organisations. Remuneration for patients’ expertise and resources also must be taken into account. There is a risk of tokenism or superficial involvement, where patient input is sought but not genuinely incorporated into decision-making processes. It is essential to address these concerns and ensure meaningful and equitable engagement and empowerment for all individuals involved (56, 61).

### 4.2 Empowering the public

A majority of practises were directed to patients. This observed bias may perhaps be grounded in the underlying fact that patients, being direct beneficiaries of healthcare services, play a central role in personalised medicine as well as the fact that genetics/genomics is not an integral part of public health (6). Efforts to integrate genomics into public health research and care should be supported and enhanced more to assess the contribution of genomics to public health. The potential of “personalised” prevention and personalised medicine is to improve individual health as well as the population health (62). In our study, the public seems more engaged in practises concerning biobanks and data-sharing topics, while being less involved in care and treatment-related practises. However, considering that the persons comprising the public may become patients themselves at certain points in their lives, it is crucial to engage the public in personalised medicine practises. The body of research already indicates that increasing genetic literacy among the general public and health professionals is crucial for facilitating the application of genomic research findings to therapeutic settings (32). Engagement practises targeting the public were often surveys as a means of assessing knowledge and opinion (49, 50). For those who participate in the less intensive engagement methods, empowerment may be stimulated through increasing awareness of personalised medicine, while the findings of such surveys may be used to improve policy. There are several pathways for citizen engagement, and concomitant ways of achieving individual empowerment, including enhancing health literacy and capacity-building. These will be stronger in the more intensive engagement methods such as the development and dissemination of education and awareness tools and materials to educate the public on genetic concepts with the use of online apps and web-based decision aids (46, 52, 63).

### 4.3 Empowering patients

As mentioned in the Introduction, patient empowerment fosters taking charge of one’s health, and entails more than just finding one’s voice (11). Patient engagement can relate to the micro (patient) level which would constitute empowerment to take control of their own care which may lead to improved health, greater satisfaction with intervention options, and better quality of life and psychological state (12, 58). As a result, there are numerous methods through which patients might be empowered. In our study, the most popular methods for engaging patients were the use of online discussions and surveys. The most intensive engagement method was the use of online/web-based decision aids; participation of patient organisation/representatives as well as app games which may presumably lead to empowerment. In order for a patient to be empowered, education, literacy and knowledge is essential. Allowing patients and patient organisations to share their perspectives on the quality of education and co-create information materials not only enhances information but can also contribute to an improvement in overall healthcare quality (64). For meaningful patient empowerment it is crucial that health care providers have adequate knowledge and skills regarding applications for personalised prevention and can communicate about these to their patients (65, 66).

In contrast, engagement can also have an impact on the macro (community) level in terms of quality of health and social services and intervention design; policy prioritisation, and cost-effectiveness (58). For instance as Perry et al. (36) and Pujol et al. (37) showed, especially patient organisations can contribute to the development of effective consent forms and other relevant information materials, highlighting the value of collaboration in decision-making processes to improve care (36, 37).

### 4.4 Lack of evaluation

It is still unknown how the identified engagement practises impact patients and the public and whether improvements result in higher-quality care. During the mapping of patient and public practises in personalised prevention, it became clear that evaluation and feedback follow-ups were frequently missing or just briefly described. This oversight creates a huge gap in the implementation of these practises, making it difficult to measure their success and make necessary modifications. Explain to patients how their feedback is used and is put into better practise is crucial to motivate patients to contribute. Furthermore, Nunn et al. (67) argue that more systematic methods of reporting and measuring involvement would be extremely valuable in developing best practises.

### 4.5 Evaluation of clinical trials

During our search we came across several clinical trials that were excluded due to the fact that no engagement was reported. Clinical studies generally do not assess patient satisfaction or public response or do not report having done so. To address this gap, it has been recommended that studies place greater emphasis on involving patients, ensuring that feedback is actively sought and incorporated (68). As well as, including the patients throughout the whole process, by actively contributing to the development of research questions before the trial takes place, which will improve trial enrolment, retention and adherence. By involving these stakeholders, clinical trials become more patient-centric, address relevant outcomes, and prioritise participant safety and wellbeing (69). This integration has the potential to improve patient outcomes, since research findings can guide tailored treatment decisions and care plans (70, 71).

### 4.6 Blurring boundaries between research and care

In the context of personalised medicine, the traditional boundaries separating research and care become less distinct (72). Several engagement practises in our study, seem to bridge the research and care domains, indicating a growing recognition of the interconnectedness between these areas. Perry et al. (36) and Appelbaum et al. (73), describe the perception among individuals that their contribution to research automatically translates into benefits for their own care, also known as therapeutic misconception. However, this belief can be misleading, posing a potential pitfall for personalised prevention. It is crucial to avoid prematurely enticing individuals to participate in research without fully informing them of the complexities involved and of the objectives (36). Embracing both domains of research and care in personalised medicine can introduce challenges, as highlighted by Day et al. (31) who pointed out that the translation of new protocols based on biological research further complicated an already complex patient pathway. By integrating the viewpoints of diverse stakeholders involved in stratified medicine, including healthcare personnel, patients, and families, the survey responses highlighted the shared high expectations for the early implementation in a London breast cancer service. Nonetheless, patients, caregivers, and staff were impacted by the new and existing forms of stratification, leading to care that was reported to frequently feel less personal rather than more personal due to the impersonal nature of the clinical interactions (31). In exploring the integration of research and care, patients’ experiences are crucial to help optimise the implementation of personalised prevention.

### 4.7 Study strengths and limitations

We utilised a thorough methodology that allowed broad-scope investigations into the landscape of public and patient engagement practises in personalised prevention and genomics. Rather than covering engagement in detail, this method allowed for a better comprehension of the range of topics/aspects relevant for understanding patient and public engagement across various domains relevant for personalised prevention. This scoping review has several limitations, as personalised prevention practises in Europe were retrieved via academic databases, practises that are not publicly available or only reported in grey literature were not included. In the literature, the terms “engagement,” “participation,” and “involvement” are frequently used interchangeably and ambiguously, with their meaning appearing self-evident. Despite calls to develop evidence-based engagement, the literature’s current lack of clear conceptualisations and definitions of engagement is a major impediment to valid measurement and analysis. We have tried to overcome this shortcoming by combining engagement in four domains which may have resulted in overlooking other relevant aspects. It is worth noting that although the search strategy employed for identifying common chronic conditions could have been expanded, we deliberately used overarching MeSH terms (e.g., neurological disorders) rather than specific subheadings (e.g., Alzheimer disease) to avoid too many unrelated search hits detailing secondary factors. However, articles may thereby be missed.

## 5 Conclusion

In conclusion, this scoping review has provided a thorough mapping of patient and public engagement practises within the context of personalised prevention in the domains of research, care, education and governance. To the best of our knowledge, this article is the first to map public and patient engagement practises in personalised prevention using genomics in Europe. The findings demonstrate the wide range of approaches and methods that can be utilised to engage patients and the general public at various stages of the empowerment and engagement spectrum, but were mostly at the lower level. It is evident that different methods are suitable for different purposes and objectives, as well as for engaging patients vs. the general public. Engaging patients and the public in personalised prevention efforts is essential to empower individuals to take an active role in their own health and wellbeing. The significance of education is evident across various facets, as it recurs across domains and is foundational for empowerment. In order for patients and the public to be empowered, education, health literacy and knowledge need to be enhanced. Moving forward, it is crucial to invest in these various possibilities and to ensure that they are continually placed prominently on the agenda. More experience and research can establish what are best models of engagement for particular goals and circumstances. By elaborating on and implementing practises that educate, engage and empower the patients and public at all levels of the engagement spectrum, we can foster a more inclusive and participatory approach to personalised prevention, ultimately leading to improved health outcomes for individuals.

## Data Availability

The original contributions presented in the study are included in the article/Supplementary material, further inquiries can be directed to the corresponding author.

## Footnotes

^1^ Embase.com
