# Supplementary material for: Engagement of patients and the public in personalised prevention in Europe using genomic information: a scoping review
Source: Front Public Health. 2024 Sep 12;12:1456853. doi: 10.3389/fpubh.2024.1456853 (PMC11427883; doi:10.3389/fpubh.2024.1456853)
Supplement: Supplementary file 2 [file Data_Sheet_2.PDF]

Supplementary Material

1 Supplementary Information full search strategies

Search strategy for OVID/Medline (28 July 2023)

Ovid MEDLINE(R) ALL <1946 to July 27, 2023>

**1**	"Citizen Science"/ or citizen-science*.ti,ab,kf.	**2,300**
**2**	exp Community-Based Participatory Research/ or exp Patients/ or exp Family/ or (public or publically or citizen* or communit* or population* or stakeholder* or stake-holder* or patient* or client* or famil* or outpatient* or inpatient* or index-case* or proband* or relative*).ti,ab,kf.	**12,382,485**
**3**	(((personalized or personalised or individual* or predictive or precision or stratif* or tailor* or targeted) adj3 preventi*) or (precision adj3 health*) or public-health-prevention or ((personalized or personalised) adj3 medicine)).ti,ab,kf.	**36,578**
**4**	2 and 3	**24,994**
**5**	1 or 4	**27,282**
**6**	exp "Consumer Health Information"/ or exp Internet/ or exp Patient Participation/ or exp "Webcasts as Topic"/ or webcast.pt. or exp Health Communication/ or exp "Surveys and Questionnaires"/ or exp Focus Groups/ or Communication/ or exp Public Opinion/ or (engagement* or intervent* or communicat* or educat* or empower* or literate* or literac* or initiative* or (ethic* adj3 legal* adj3 social*) or inform* or misinform* or consult* or involve* or collaborat* or dialogue* or internet or web-contest* or forum* or fora or capacity-building or workshop* or meeting* or website* or newsletter* or news-letter* or fact-sheet* or factsheet* or web-2-0* or social-media* or twitter* or instagram or tiktok* or tik-tok* or facebook or face-book or platform* or podcast* or pod-cast* or channel* or youtube or feeds or reference-group* or joint-plan* or blog* or bulletin* or circular* or social-network* or open-discussion* or digital-tool* or design-thinking* or public-action* or (concept* adj3 (framework* or frame-work*)) or public-participati* or patient-participati* or (online adj3 aware*) or survey* or focus-group* or questionnaire*).ti,ab,kf.	**8,151,032**
**7**	exp Genetics/ or exp Genetic Testing/ or exp Genetics, Population/ or exp Genetic Services/ or exp "Genetic Predisposition to Disease"/ or exp Genetic Counseling/ or exp Genetic Privacy/ or Genetics/ or exp Genetic Research/ or exp Human Genetics/ or Genomics/ or exp Pharmacogenetics/ or (genomic* or genetic* or pharmacogenetic*).ti,ab,kf.	**1,749,819**
**8**	Neoplasms/ or Myelodysplastic Syndromes/ or (tumor* or tumour* or cancer* or malignan* or carcinogen* or neoplas* or oncogen* or carcinoma* or oncolog*).ti,ab,kf.	**4,208,264**
**9**	Cardiovascular Physiological Phenomena/ or Cardiovascular Diseases/ or exp Stroke/ or (cardiovascul* or cardio-vascul* or cvd*1).ti,ab,kf.	**784,248**
**10**	Neurodegenerative Diseases/ or (neurodegenerative-disease* or neurologic-degenerative-disease* or degenerative-neurologic-disease* or nervous-system-degenerative-disease* or neurodegenerative-disorder* or neurologic-degenerative-condition* or degenerative-neurologic-disorder* or spinal-cord-degenerative-disease*).ti,ab,kf.	**106,770**

**11**	8 or 9 or 10	**5,018,736**
**12**	5 and 6 and 7 and 11	**1,744**
**13**	12 not (exp Animals/ not exp Humans/)	**1,733**
**14**	limit 13 to yr="2015 -Current"	**1,205**

Search strategy for Embase.com (28 August 2023)

No.	Query	Results
**#16**	#15 AND [2015-2023]/py	**3,071**
**#15**	#14 NOT ([animals]/lim NOT [humans]/lim)	**3,988**
**#14**	#12 NOT #13	**4,014**
**#13**	#12 AND ('Conference Abstract'/it OR 'Conference Paper'/it OR 'Conference Review'/it)	**2,393**
**#12**	#5 AND #6 AND #7 AND #11	**6,407**
**#11**	#8 OR #9 OR #10	**7,086,760**
**#10**	'degenerative disease'/de OR 'neurodegenerative disease*':ti,ab,kw OR 'neurologic degenerative disease*':ti,ab,kw OR 'degenerative neurologic disease*':ti,ab,kw OR 'nervous system degenerative disease*':ti,ab,kw OR 'neurodegenerative disorder*':ti,ab,kw OR 'neurologic degenerative condition*':ti,ab,kw OR 'degenerative neurologic disorder*':ti,ab,kw OR 'spinal cord degenerative disease*':ti,ab,kw	**160,607**
**#9**	'cardiovascular function'/de OR 'cardiovascular disease'/de OR 'cerebrovascular accident'/de OR cardiovascul*:ti,ab,kw OR 'cardio vascul*':ti,ab,kw OR cvd*1:ti,ab,kw	**1,291,696**
**#8**	'neoplasm'/de OR 'myelodysplastic syndrome'/de OR tumor*:ti,ab,kw OR tumour*:ti,ab,kw OR cancer*:ti,ab,kw OR malignan*:ti,ab,kw OR carcinogen*:ti,ab,kw OR neoplas*:ti,ab,kw OR oncogen*:ti,ab,kw OR carcinoma*:ti,ab,kw OR oncolog*:ti,ab,kw	**5,777,822**
**#7**	'genetics'/exp OR 'genetic screening'/exp OR 'population genetics'/exp OR 'genetic service'/exp OR 'genetic predisposition'/exp OR 'genetic counseling'/exp OR 'genetic privacy'/exp OR 'genomics'/exp OR 'pharmacogenetics'/exp OR genomic*:ti,ab,kw OR genetic*:ti,ab,kw OR pharmacogenetic*:ti,ab,kw	**2,910,744**
**#6**	'consumer health information'/exp OR 'internet'/exp OR 'patient participation'/exp OR 'mass communication'/exp OR 'medical information'/exp OR 'questionnaire'/exp OR 'interpersonal communication'/exp OR 'public opinion'/exp OR engagement*:ti,ab,kw OR intervent*:ti,ab,kw OR communicat*:ti,ab,kw OR educat*:ti,ab,kw OR empower*:ti,ab,kw OR literate*:ti,ab,kw OR literac*:ti,ab,kw OR initiative*:ti,ab,kw OR ((ethic* NEAR/3 legal* NEAR/3 social*):ti,ab,kw) OR inform*:ti,ab,kw OR misinform*:ti,ab,kw OR consult*:ti,ab,kw OR involve*:ti,ab,kw OR collaborat*:ti,ab,kw OR dialogue*:ti,ab,kw OR internet:ti,ab,kw OR 'web contest*':ti,ab,kw OR forum*:ti,ab,kw OR fora:ti,ab,kw OR 'capacity building':ti,ab,kw OR workshop*:ti,ab,kw OR meeting*:ti,ab,kw OR website*:ti,ab,kw OR newsletter*:ti,ab,kw OR 'news letter*':ti,ab,kw OR 'fact sheet*':ti,ab,kw OR factsheet*:ti,ab,kw OR 'web 2 0*':ti,ab,kw OR 'social media*':ti,ab,kw OR twitter*:ti,ab,kw OR instagram:ti,ab,kw OR tiktok*:ti,ab,kw	**10,760,119**

	OR 'tik tok*':ti,ab,kw OR facebook:ti,ab,kw OR 'face book':ti,ab,kw OR platform*:ti,ab,kw OR podcast*:ti,ab,kw OR 'pod cast*':ti,ab,kw OR channel*:ti,ab,kw OR youtube:ti,ab,kw OR feeds:ti,ab,kw OR 'reference group*':ti,ab,kw OR 'joint plan*':ti,ab,kw OR blog*:ti,ab,kw OR bulletin*:ti,ab,kw OR circular*:ti,ab,kw OR 'social network*':ti,ab,kw OR 'open discussion*':ti,ab,kw OR 'digital tool*':ti,ab,kw OR 'design thinking*':ti,ab,kw OR 'public action*':ti,ab,kw OR ((concept* NEAR/3 (framework* OR 'frame work*')):ti,ab,kw) OR 'public participati*':ti,ab,kw OR 'patient participati*':ti,ab,kw OR ((online NEAR/3 aware*):ti,ab,kw) OR survey*:ti,ab,kw OR 'focus group*':ti,ab,kw OR questionnaire*:ti,ab,kw	
**#5**	#1 OR #4	**71,205**
**#4**	#2 AND #3	**69,241**
**#3**	'personalized medicine'/exp OR (((personalized OR personalised OR individual* OR predictive OR precision OR stratif* OR tailor* OR targeted) NEAR/3 preventi*):ti,ab,kw) OR ((precision NEAR/3 health*):ti,ab,kw) OR 'public health prevention':ti,ab,kw OR (((personalized OR personalised) NEAR/3 medicine):ti,ab,kw)	**103,337**
**#2**	'participatory research'/exp OR 'patient'/exp OR 'family'/exp OR public:ti,ab,kw OR publically:ti,ab,kw OR citizen*:ti,ab,kw OR communit*:ti,ab,kw OR population*:ti,ab,kw OR stakeholder*:ti,ab,kw OR 'stake holder*':ti,ab,kw OR patient*:ti,ab,kw OR client*:ti,ab,kw OR famil*:ti,ab,kw OR outpatient*:ti,ab,kw OR inpatient*:ti,ab,kw OR 'index case*':ti,ab,kw OR proband*:ti,ab,kw OR relative*:ti,ab,kw	**17,099,954**
**#1**	'citizen science'/exp OR 'citizen science*':ti,ab,kw	**1,987**

Search strategy for Elsevier/Scopus (28 August 2023)

History Count	Search Terms	Results
**9**	#8 AND PUBYEAR > 2015-2023	**1,371**
**7**	#3 AND #4 AND #5 AND #6	**2,045**
**6**	TITLE-ABS (engagement* OR intervent* OR communicat* OR educat* OR empower* OR literate* OR literac* OR initiative* OR (ethic* W/3 legal* W/3 social*) OR inform* OR misinform* OR consult* OR involve* OR collaborat* OR dialogue* OR internet OR web-contest* OR forum* OR fora OR capacity-building OR workshop* OR meeting* OR website* OR newsletter* OR news-letter* OR fact-sheet* OR factsheet* OR web-2-0* OR social-media* OR twitter* OR instagram OR tiktok* OR tik-tok* OR facebook OR face-book OR platform* OR podcast* OR pod-cast* OR channel* OR youtube OR feeds OR reference-group* OR joint-plan* OR blog* OR bulletin* OR circular* OR social-network* OR open-discussion* OR digital-tool* OR design-thinking* OR public-action* OR (concept* W/3 (framework* OR frame-work*)) OR public-participati* OR patient-participati* OR (online W/3 aware*) OR survey* OR focus-group* OR questionnaire*) OR AUTHKEY (engagement* OR intervent* OR communicat* OR educat* OR empower* OR literate* OR literac* OR initiative* OR (ethic* W/3 legal* W/3 social*) OR inform* OR misinform* OR consult* OR involve* OR collaborat* OR dialogue* OR internet OR web-contest* OR forum* OR fora OR capacity-building OR workshop* OR meeting* OR website* OR newsletter* OR news-letter* OR fact-sheet* OR factsheet* OR web-2-0* OR social-media* OR twitter* OR instagram OR tiktok* OR tik-tok* OR facebook OR	**20,405,004**

	face-book OR platform* OR podcast* OR pod-cast* OR channel* OR youtube OR feeds OR reference-group* OR joint-plan* OR blog* OR bulletin* OR circular* OR social-network* OR open-discussion* OR digital-tool* OR design-thinking* OR public-action* OR (concept* W/3 (framework* OR frame-work*)) OR public-participati* OR patient-participati* OR (online W/3 aware*) OR survey* OR focus-group* OR questionnaire*)	
**5**	(TITLE-ABS (tumor* OR tumour* OR cancer* OR malignan* OR carcinogen* OR neoplas* OR oncogen* OR carcinoma* OR oncolog*) OR AUTHKEY (tumor* OR tumour* OR cancer* OR malignan* OR carcinogen* OR neoplas* OR oncogen* OR carcinoma* OR oncolog*)) OR (TITLE-ABS (cardiovascul* OR cardio-vascul* OR cvd*) OR AUTHKEY (cardiovascul* OR cardio-vascul* OR cvd*)) OR (TITLE-ABS (neurodegenerative-disease* OR neurologic-degenerative-disease* OR degenerative-neurologic-disease* OR nervous-system-degenerative-disease* OR neurodegenerative-disorder* OR neurologic-degenerative-condition* OR degenerative-neurologic-disorder* OR spinal-cord-degenerative-disease*) OR AUTHKEY (neurodegenerative-disease* OR neurologic-degenerative-disease* OR degenerative-neurologic-disease* OR nervous-system-degenerative-disease* OR neurodegenerative-disorder* OR neurologic-degenerative-condition* OR degenerative-neurologic-disorder* OR spinal-cord-degenerative-disease*))	**5,748,587**
**4**	TITLE-ABS (genomic* OR genetic* OR pharmacogenetic*) OR AUTHKEY (genomic* OR genetic* OR pharmacogenetic*)	**2,250,453**
**3**	#1 OR #2	**43,611**
**2**	(TITLE-ABS (public OR publically OR citizen* OR communit* OR population* OR stakeholder* OR stake-holder* OR patient* OR client* OR famil* OR outpatient* OR inpatient* OR index-case* OR proband* OR relative*) OR AUTHKEY (public OR publically OR citizen* OR communit* OR population* OR stakeholder* OR stake-holder* OR patient* OR client* OR famil* OR outpatient* OR inpatient* OR index-case* OR proband* OR relative*)) AND (TITLE-ABS ((personalized OR personalised OR individual* OR predictive OR precision OR stratif* OR tailor* OR targeted) W/3 (preventi*) OR (precision W/3 health*) OR public-health-prevention OR ((personalized OR personalised) W/3 medicine)) OR AUTHKEY ((personalized OR personalised OR individual* OR predictive OR precision OR stratif* OR tailor* OR targeted) W/3 (preventi*) OR (precision W/3 health*) OR public-health-prevention OR ((personalized OR personalised) W/3 medicine)))	**34,439**
**1**	TITLE-ABS (citizen-science*) OR AUTHKEY (citizen-science*)	**9,191**

Search strategy for Clarivate Analytics/Web of Science Core Collection (28 August 2023)

5	#4 AND #3 AND #2 AND #1<br>Refined by years: 2015 or 2016 or 2017 or 2018 or 2019 or 2020 or 2021 or 2022 or 2023	**1,556**
4	TS=("tumor*" OR "tumour*" OR "cancer*" OR "malignan*" OR "carcinogen*" OR "neoplas*" OR "oncogen*" OR "carcinoma*" OR "oncolog*" OR "cardiovascul*" OR "cardio-vascul*" OR "cvd*" OR "neurodegenerative-disease*" OR "neurologic-degenerative-disease*" OR "degenerative-neurologic-disease*" OR "nervous-system-degenerative-disease*" OR "neurodegenerative-disorder*" OR "neurologic-	**5,776,990**

	degenerative-condition*" OR "degenerative-neurologic-disorder*" OR "spinal-cord-degenerative-disease*")	
3	TS=("genomic*" OR "genetic*" OR "pharmacogenetic*")	**1,977,230**
2	TS=("engagement*" OR "intervent*" OR "communicat*" OR "educat*" OR "empower*" OR "literate*" OR "literac*" OR "initiative*" OR ("ethic*" NEAR/3 "legal*" NEAR/3 "social*") OR "inform*" OR "misinform*" OR "consult*" OR "involve*" OR "collaborat*" OR "dialogue*" OR "internet" OR "web-contest*" OR "forum*" OR "fora" OR "capacity-building" OR "workshop*" OR "meeting*" OR "website*" OR "newsletter*" OR "news-letter*" OR "fact-sheet*" OR "factsheet*" OR "web-2-0*" OR "social-media*" OR "twitter*" OR "instagram" OR "tiktok*" OR "tik-tok*" OR "facebook" OR "face-book" OR "platform*" OR "podcast*" OR "pod-cast*" OR "channel*" OR "youtube" OR "feeds" OR "reference-group*" OR "joint-plan*" OR "blog*" OR "bulletin*" OR "circular*" OR "social-network*" OR "open-discussion*" OR "digital-tool*" OR "design-thinking*" OR "public-action*" OR ("concept*" NEAR/3 ("framework*" OR "frame-work*")) OR "public-participati*" OR "patient-participati*" OR ("online" NEAR/3 "aware*") OR "survey*" OR "focus-group*" OR "questionnaire*")	**13,170,977**
1	TS=("citizen-science*" OR ("public" OR "publically" OR "citizen*" OR "communit*" OR "population*" OR "stakeholder*" OR "stake-holder*" OR "patient*" OR "client*" OR "famil*" OR "outpatient*" OR "inpatient*" OR "index-case*" OR "proband*" OR "relative*") AND (("personalized" OR "personalised" OR "individual*" OR "predictive" OR "precision" OR "stratif*" OR "tailor*" OR "targeted") NEAR/3 ("preventi*") OR ("precision" NEAR/3 "health*") OR "public-health-prevention" OR (("personalized" OR "personalised") NEAR/3 "medicine")))	**37,567**

Search strategy for Ebsco/APA PsycINFO (28 August 2023)

#	Query	Limiters/ Expanders	Results
**S6**	S1 AND S2 AND S3 AND S4	Limiters - Publication Year: 2015-2023	**76**
**S5**	S1 AND S2 AND S3 AND S4		**110**
**S4**	DE "Neoplasms" OR TI(tumor* OR tumour* OR cancer* OR malignan* OR carcinogen* OR neoplas* OR oncogen* OR carcinoma* OR oncolog*) OR AB(tumor* OR tumour* OR cancer* OR malignan* OR carcinogen* OR neoplas* OR oncogen* OR carcinoma* OR oncolog*) OR KW(tumor* OR tumour* OR cancer* OR malignan* OR carcinogen* OR neoplas* OR oncogen* OR carcinoma* OR oncolog*) OR DE "Cardiovascular Health" OR DE "Cardiovascular Disorders" OR DE "Cerebrovascular Accidents" OR TI(cardiovascul* OR cardio-vascul* OR cvd*) OR AB(cardiovascul* OR cardio-vascul* OR cvd*) OR KW(cardiovascul* OR cardio-vascul* OR cvd*) OR DE "Neurodegenerative Diseases" OR TI(neurodegenerative-disease* OR neurologic-degenerative-disease* OR degenerative-neurologic-disease* OR nervous-system-degenerative-disease* OR neurodegenerative-disorder* OR neurologic-degenerative-condition* OR degenerative-neurologic-disorder* OR spinal-cord-degenerative-disease*) OR AB(neurodegenerative-disease* OR neurologic-degenerative-disease* OR degenerative-neurologic-disease* OR		**177,177**

	nervous-system-degenerative-disease* OR neurodegenerative-disorder* OR neurologic-degenerative-condition* OR degenerative-neurologic-disorder* OR spinal-cord-degenerative-disease*) OR KW(neurodegenerative-disease* OR neurologic-degenerative-disease* OR degenerative-neurologic-disease* OR nervous-system-degenerative-disease* OR neurodegenerative-disorder* OR neurologic-degenerative-condition* OR degenerative-neurologic-disorder* OR spinal-cord-degenerative-disease*)		
**S 3**	DE "Genome" OR DE "Genomic Sequencing" OR DE "Genetics" OR DE "Behavioral Genetics" OR DE "Genetic Engineering" OR DE "Genetic Processes" OR DE "Genomics" OR DE "Pharmacogenetics" OR DE "Population Genetics" OR DE "Gene Expression" OR DE "Mutations" OR DE "Polymorphism" OR DE "Genetic Testing" OR DE "Genetic Counseling" OR TI(genomic* OR genetic* OR pharmacogenetic*) OR AB(genomic* OR genetic* OR pharmacogenetic*) OR KW(genomic* OR genetic* OR pharmacogenetic*)		**156,166**
**S 2**	DE "Digital Health Resources" OR DE "Digital Mental Health Resources" OR DE "Health Information" OR DE "Electronic Collaboration" OR DE "Social Media" OR DE "Online Social Networks" OR DE "Websites" OR DE "Blog" OR DE "Internet" OR DE "Client Participation" OR DE "Surveys" OR DE "Consumer Surveys" OR DE "Mail Surveys" OR DE "Online Surveys" OR DE "Telephone Surveys" OR DE "Questionnaires" OR DE "Communication" OR DE "Written Communication" OR DE "Oral Communication" OR DE "Verbal Communication" OR DE "Public Opinion" OR TI(engagement* OR intervent* OR communicat* OR educat* OR empower* OR literate* OR literac* OR initiative* OR (ethic* N3 legal* N3 social*) OR inform* OR misinform* OR consult* OR involve* OR collaborat* OR dialogue* OR internet OR web-contest* OR forum* OR fora OR capacity-building OR workshop* OR meeting* OR website* OR newsletter* OR news-letter* OR fact-sheet* OR factsheet* OR web-2-0* OR social-media* OR twitter* OR instagram OR tiktok* OR tik-tok* OR facebook OR face-book OR platform* OR podcast* OR pod-cast* OR channel* OR youtube OR feeds OR reference-group* OR joint-plan* OR blog* OR bulletin* OR circular* OR social-network* OR open-discussion* OR digital-tool* OR design-thinking* OR public-action* OR (concept* N3 (framework* OR frame-work*)) OR public-participati* OR patient-participati* OR (online N3 aware*) OR survey* OR focus-group* OR questionnaire*) OR AB(engagement* OR intervent* OR communicat* OR educat* OR empower* OR literate* OR literac* OR initiative* OR (ethic* N3 legal* N3 social*) OR inform* OR misinform* OR consult* OR involve* OR collaborat* OR dialogue* OR internet OR web-contest* OR forum* OR fora OR capacity-building OR workshop* OR meeting* OR website* OR newsletter* OR news-letter* OR fact-sheet* OR factsheet* OR web-2-0* OR social-media* OR twitter* OR instagram OR tiktok* OR tik-tok* OR facebook OR face-book OR platform* OR podcast* OR pod-cast* OR channel* OR youtube OR feeds OR reference-group* OR joint-plan* OR blog* OR bulletin* OR circular* OR social-network* OR open-discussion* OR digital-tool* OR design-thinking* OR public-action* OR (concept* N3 (framework* OR frame-work*)) OR public-participati* OR patient-participati* OR (online N3 aware*) OR survey* OR focus-group* OR questionnaire*) OR KW(engagement* OR intervent* OR communicat* OR educat* OR empower* OR literate* OR literac* OR initiative* OR (ethic* N3 legal*		**2,593,153**

	N3 social*) OR inform* OR misinform* OR consult* OR involve* OR collaborat* OR dialogue* OR internet OR web-contest* OR forum* OR fora OR capacity-building OR workshop* OR meeting* OR website* OR newsletter* OR news-letter* OR fact-sheet* OR factsheet* OR web-2-0* OR social-media* OR twitter* OR instagram OR tiktok* OR tik-tok* OR facebook OR face-book OR platform* OR podcast* OR pod-cast* OR channel* OR youtube OR feeds OR reference-group* OR joint-plan* OR blog* OR bulletin* OR circular* OR social-network* OR open-discussion* OR digital-tool* OR design-thinking* OR public-action* OR (concept* N3 (framework* OR frame-work*)) OR public-participati* OR patient-participati* OR (online N3 aware*) OR survey* OR focus-group* OR questionnaire*)		
**S 1**	TI(citizen-science*) OR AB(citizen-science*) OR KW(citizen-science*) OR (DE "Patients" OR DE "Geriatric Patients" OR DE "Hospitalized Patients" OR DE "Medical Patients" OR DE "Outpatients" OR DE "Patient Safety" OR DE "Psychiatric Patients" OR DE "Surgical Patients" OR DE "Terminally Ill Patients" OR DE "Family" OR DE "Biological Family" OR DE "Family Members" OR DE "Family Relations" OR TI(public OR publically OR citizen* OR communit* OR population* OR stakeholder* OR stake-holder* OR patient* OR client* OR famil* OR outpatient* OR inpatient* OR index-case* OR proband* OR relative*) OR AB(public OR publically OR citizen* OR communit* OR population* OR stakeholder* OR stake-holder* OR patient* OR client* OR famil* OR outpatient* OR inpatient* OR index-case* OR proband* OR relative*) OR KW(public OR publically OR citizen* OR communit* OR population* OR stakeholder* OR stake-holder* OR patient* OR client* OR famil* OR outpatient* OR inpatient* OR index-case* OR proband* OR relative*)) AND (DE "Precision Medicine" OR TI((personalized OR personalised OR individual* OR predictive OR precision OR stratif* OR tailor* OR targeted) N3 (preventi*) OR (precision N3 health*) OR public-health-prevention OR ((personalized OR personalised) N3 medicine)) OR AB((personalized OR personalised OR individual* OR predictive OR precision OR stratif* OR tailor* OR targeted) N3 (preventi*) OR (precision N3 health*) OR public-health-prevention OR ((personalized OR personalised) N3 medicine)) OR KW((personalized OR personalised OR individual* OR predictive OR precision OR stratif* OR tailor* OR targeted) N3 (preventi*) OR (precision N3 health*) OR public-health-prevention OR ((personalized OR personalised) N3 medicine)))		**5,758**

Search strategy for ProQuest/International Bibliography of Social Sciences (30 August 2023)

S2	(MAINSUBJECT.EXACT.EXPLODE("Engagement") OR MAINSUBJECT.EXACT.EXPLODE("Communication") OR MAINSUBJECT.EXACT("Education") OR MAINSUBJECT.EXACT.EXPLODE("Empowerment") OR MAINSUBJECT.EXACT("Health information") OR MAINSUBJECT.EXACT.EXPLODE("Information transfer") OR MAINSUBJECT.EXACT("Access to information") OR MAINSUBJECT.EXACT.EXPLODE("Information sharing") OR MAINSUBJECT.EXACT.EXPLODE("Internet") OR MAINSUBJECT.EXACT.EXPLODE("Social media") OR MAINSUBJECT.EXACT.EXPLODE("Reference groups") OR MAINSUBJECT.EXACT.EXPLODE("Patient participation") OR MAINSUBJECT.EXACT.EXPLODE("Polls & surveys") OR MAINSUBJECT.EXACT.EXPLODE("Focus groups") OR MAINSUBJECT.EXACT.EXPLODE("Questionnaires")) OR noft(("engagement*" OR	1546708

	"intervent*" OR "communicat*" OR "educat*" OR "empower*" OR "literate*" OR "literac*" OR "initiative*" OR ("ethic*" NEAR/3 "legal*" NEAR/3 "social*") OR "inform*" OR "misinform*" OR "consult*" OR "involve*" OR "collaborat*" OR "dialogue*" OR "internet" OR "web-contest*" OR "forum*" OR "fora" OR "capacity-building" OR "workshop*" OR "meeting*" OR "website*" OR "newsletter*" OR "news-letter*" OR "fact-sheet*" OR "factsheet*" OR "web-2-0*" OR "social-media*" OR "twitter*" OR "instagram" OR "tiktok*" OR "tik-tok*" OR "facebook" OR "face-book" OR "platform*" OR "podcast*" OR "pod-cast*" OR "channel*" OR "youtube" OR "feeds" OR "reference-group*" OR "joint-plan*" OR "blog*" OR "bulletin*" OR "circular*" OR "social-network*" OR "open-discussion*" OR "digital-tool*" OR "design-thinking*" OR "public-action*" OR ("concept*" NEAR/3 ("framework*" OR "frame-work*")) OR "public-participati*" OR "patient-participati*" OR ("online" NEAR/3 "aware*") OR "survey*" OR "focus-group*" OR "questionnaire*"))	
S3	noft(("citizen-science*" OR ("public" OR "publically" OR "citizen*" OR "communit*" OR "population*" OR "stakeholder*" OR "stake-holder*" OR "patient*" OR "client*" OR "famil*" OR "outpatient*" OR "inpatient*" OR "index-case*" OR "proband*" OR "relative*") AND (("personalized" OR "personalised" OR "individual*" OR "predictive" OR "precision" OR "stratif*" OR "tailor*" OR "targeted") NEAR/3 ("preventi*") OR ("precision" NEAR/3 "health*") OR "public-health-prevention" OR (("personalized" OR "personalised") NEAR/3 "medicine"))))	1263
S4	MAINSUBJECT.EXACT.EXPLODE("Genetic disorders") OR MAINSUBJECT.EXACT.EXPLODE("Pharmacogenetics") OR MAINSUBJECT.EXACT.EXPLODE("Medical genetics") OR MAINSUBJECT.EXACT.EXPLODE("Psychiatric genetics") OR MAINSUBJECT.EXACT.EXPLODE("Genetics") OR MAINSUBJECT.EXACT.EXPLODE("Genetic counseling") OR MAINSUBJECT.EXACT.EXPLODE("Genetic testing") OR MAINSUBJECT.EXACT.EXPLODE("Genetic family histories") OR ("genomic*" OR "genetic*" OR "pharmacogenetic*")	69455
S5	MAINSUBJECT.EXACT("Cancer") OR MAINSUBJECT.EXACT("Neurodegenerative diseases") OR MAINSUBJECT.EXACT("Cardiovascular diseases") OR ("tumor*" OR "tumour*" OR "cancer*" OR "malignan*" OR "carcinogen*" OR "neoplas*" OR "oncogen*" OR "carcinoma*" OR "oncolog*" OR "cardiovascul*" OR "cardio-vascul*" OR "cvd*" OR "neurodegenerative-disease*" OR "neurologic-degenerative-disease*" OR "degenerative-neurologic-disease*" OR "nervous-system-degenerative-disease*" OR "neurodegenerative-disorder*" OR "neurologic-degenerative-condition*" OR "degenerative-neurologic-disorder*" OR "spinal-cord-degenerative-disease*")	55226
S6	[S2] AND [S3] AND [S4] AND [S5]	56
S7	([S2] AND [S3] AND [S4] AND [S5]) AND pd(20150101-20230831)	38
